# Estimated number and percentage of US adults with atherosclerotic cardiovascular disease recommended add-on lipid-lowering therapy by the 2018 AHA/ACC multi-society cholesterol guideline

**DOI:** 10.1016/j.ahjo.2022.100201

**Published:** 2022-08-27

**Authors:** Chibuike J. Alanaeme, Vera Bittner, Todd M. Brown, Lisandro D. Colantonio, Nafeesa Dhalwani, Jenna Jones, Bethany Kalich, Jason Exter, Elizabeth A. Jackson, Emily B. Levitan, Bharat Poudel, Zhixin Wang, Mark Woodward, Paul Muntner, Robert S. Rosenson

**Affiliations:** a Department of Epidemiology, University of Alabama at Birmingham, Birmingham, AL, USA; b Department of Medicine, Division of Cardiovascular Disease, University of Alabama at Birmingham, Birmingham, AL, USA; c Center for Observational Research, Amgen Inc., Thousand Oaks, CA, USA; d Amgen Inc., Thousand Oaks, CA, USA; e The George Institute for Global Health, Imperial College London, UK; f The George Institute for Global Health, University of New South Wales, Sydney, Australia; g Mount Sinai Heart, Icahn School of Medicine at Mount Sinai, New York, NY, USA

**Keywords:** Add-on lipid-lowering therapy, AHA/ACC, ASCVD, Cholesterol, Ezetimibe, Lipid, PCSK9i

## Abstract

**Study objective:**

The 2018 American Heart Association/American College of Cardiology (AHA/ACC) cholesterol guideline recommends a maximally-tolerated statin with add-on lipid-lowering therapy, ezetimibe and/or proprotein convertase subtilisin/kexin type 9 (PCSK9) for adults with very-high atherosclerotic cardiovascular disease (ASCVD) risk to achieve a low-density lipoprotein cholesterol (LDL-C) <70 mg/dL. We estimated the percentage of US adults with ASCVD recommended, by the 2018 AHA/ACC cholesterol guideline, and receiving add-on lipid-lowering therapy.

**Design, setting, and participants:**

Cross-sectional study including 805 participants from the US National Health and Nutrition Examination Survey (NHANES) 2013–2020 data. NHANES sampling weights were used to obtain estimates for the US adult population.

**Main measures:**

Very-high ASCVD risk was defined as either: ≥2 ASCVD events, or one ASCVD event with ≥2 high-risk conditions. Being recommended add-on lipid-lowering therapy was defined as having very-high ASCVD risk and LDL-C ≥ 70 mg/dL, or LDL-C < 70 mg/dL while taking ezetimibe or a PCSK9 inhibitor.

**Results:**

An estimated 18.7 (95%CI, 16.0–21.4) million US adults had ASCVD, of whom 81.6 % (95%CI, 76.7 %–86.4 %) had very-high ASCVD risk, and 60.1 % (95%CI, 54.5 %–65.7 %) had very-high ASCVD risk and LDL-C ≥ 70 mg/dL. Overall, 61.4 % (95%CI, 55.8 %–66.9 %) were recommended add-on lipid-lowering therapy and 3.2 % (95 % CI, 1.2 %–5.3 %) were taking it. Smokers, adults with diabetes, hypertension and chronic kidney disease were more likely, while those taking atorvastatin or rosuvastatin were less likely, to be recommended add-on lipid-lowering therapy.

**Conclusion:**

The majority of US adults with ASCVD are recommended add-on lipid-lowering therapy by the 2018 AHA/ACC cholesterol guideline but few are receiving it.

## 1 Introduction

The 2018 American Heart Association (AHA) and American College of Cardiology (ACC) multi-society cholesterol guideline recommends statin therapy for all adults with a history of atherosclerotic cardiovascular disease (ASCVD) to reduce the risk of a recurrent ASCVD event [1]. A high-intensity statin is recommended for adults <75 years of age, and a moderate or high-intensity statin is recommended for adults ≥75 years of age [1]. Add-on lipid-lowering therapy with ezetimibe and/or a proprotein convertase subtilisin/kexin type 9 (PCSK9) inhibitor is recommended for adults who have experienced an ASCVD event, have a very high risk for a recurrent ASCVD event, and have low-density lipoprotein cholesterol (LDL-C) ≥ 70 mg/dL despite taking a maximally tolerated statin [1].

In previous studies of adults with ASCVD, those with very-high ASCVD risk according to the 2018 AHA/ACC cholesterol guideline had 2 to 3 times higher risk for recurrent ASCVD events compared to those without very-high ASCVD risk [2], [3]. Furthermore, prior studies using administrative claims data for US Veterans and adults with commercial or government health insurance suggest that a low percentage of adults with very-high ASCVD risk are taking ezetimibe or a PCSK9 inhibitor [3], [4], [5]. The percentage of adults with very-high ASCVD risk recommended and taking these medications among a general US population sample is unclear. Therefore, we estimated the number and percentage of US adults with ASCVD recommended for add-on lipid-lowering therapy with ezetimibe or a PCSK9 inhibitor by the 2018 AHA/ACC cholesterol guideline. Additionally, we estimated the number and percentage of adults recommended for add-on lipid-lowering therapy who were taking ezetimibe or a PCSK9 inhibitor.

## 2 Methods

### 2.1 Participant identification and study population

We conducted a cross-sectional study using data from the US National Health and Nutrition Examination Survey (NHANES). NHANES was performed by the National Center for Health Statistics of the Centers for Disease Control and Prevention (CDC) and has been conducted in 2-year cycles since 1999–2000 [6]. Due to the coronavirus pandemic, NHANES suspended field operations in March 2020 and combined the 2017–2018 and 2019–2020 cycles to create a 2017 - March 2020 pre-pandemic cycle [7]. To provide stable nationally representative prevalence estimates, we pooled data from the three most recent cycles of NHANES: 2013–2014, 2015–2016, and 2017-March 2020 (N = 35,706). We restricted the analysis to participants who completed the NHANES interview and examination, were ≥20 years of age, attended the morning examination, and fasted for 8 to 24 hours before their study visit. We also restricted the sample to participants with a history of ASCVD, defined as self-reported coronary heart disease (CHD), myocardial infarction (MI), or stroke. The sample was further restricted to participants who had a complete information on triglycerides, total cholesterol, and high-density lipoprotein cholesterol (HDL—C). After applying these criteria, 805 participants were included in all analyses (Supplemental Fig. 1).

### 2.2 Data collection

NHANES collected demographic and health-related data during an in-home interview and a study visit conducted in a mobile examination center. During the interview, self-reported information was collected on age, sex, race/ethnicity, smoking status, a prior diagnosis of diabetes, CHD, MI, stroke, heart failure, and glucose-lowering and antihypertensive medications. Additional information on medication use was determined by asking participants to provide containers for the prescription medications they used in the past 30 days. During the study visit, trained staff measured blood pressure and collected urine and blood specimens. Of relevance to the current study, the blood was used to measure glycated hemoglobin, serum creatinine, triglycerides, total cholesterol, and HDL—C. Using the urine sample, albumin and creatinine were measured. Chronic kidney disease (CKD) was defined as an estimated glomerular filtration rate (eGFR) < 60 mL/min/1.73 m^2^ or albumin-to-creatinine ratio > 30 mg/g. Diabetes was defined as a glycated hemoglobin level ≥ 6.5 % or a self-reported history of diabetes with the concurrent use of insulin or oral glucose-lowering medication. We defined hypertension as an average systolic blood pressure ≥ 130 mmHg or an average diastolic blood pressure ≥ 80 mmHg or self-reported history of hypertension with current use of antihypertensive medication. We used the Sampson equation to estimate LDL-C and defined a persistently elevated LDL-C as ≥100 mg/dL [8]. The use of statins, ezetimibe, and PCSK9 inhibitors was identified through medication container review. Statin dosages were not available in NHANES. Participants were categorized as not taking a statin, taking atorvastatin or rosuvastatin, or taking other statins including simvastatin, fluvastatin, pitavastatin, pravastatin, or lovastatin. Atorvastatin and rosuvastatin were considered in a separate category as they are available in high-intensity dosages.

### 2.3 Definitions of very-high ASCVD risk and AHA/ACC recommendation

The 2018 AHA/ACC cholesterol guideline defines very-high ASCVD risk as having a history of ≥2 ASCVD events or having a history of one ASCVD event with at least 2 high-risk conditions (Supplemental Table 1). The guideline recommends considering add-on lipid-lowering therapy with ezetimibe and/or a PCSK9 inhibitor for adults with very-high ASCVD risk and LDL-C level ≥ 70 mg/dL while taking a maximally tolerated statin dosage [1]. As data were not available on whether NHANES participants had experienced statin-associated muscle symptoms, or were taking a maximally tolerated statin intensity, we considered those with very-high ASCVD risk and LDL-C level ≥ 70 mg/dL, regardless of statin use or dosage, as being recommended for add-on lipid-lowering therapy. Also, we considered participants with very-high ASCVD risk and LDL-C < 70 mg/dL while taking ezetimibe or a PCSK9 inhibitor as being recommended for add-on lipid-lowering therapy.

### 2.4 Statistical analysis

NHANES did not collect information on the number of MIs or strokes that participants had experienced. Therefore, we used data from adults with health insurance in the Medicare and MarketScan (Truven Health Analytics, IBM Watson Health, Ann Arbor, Michigan) databases to impute a history of ≥2 MIs or stroke events in NHANES. Details on the populations in the Medicare and MarketScan databases are provided in the Supplemental Methods. We appended data from Medicare and MarketScan to the NHANES database and used multiple imputation with 50 datasets to impute having ≥2 ASCVD events among NHANES participants with ASCVD based on the calendar year, age, sex, diabetes, hypertension, CKD, smoking status, heart failure, and statin use. To validate this approach, we appended the Medicare and MarketScan data to baseline data from the Reasons for Geographic And Racial Differences in Stroke (REGARDS) study, a prospective cohort study that collected self-reported information on having ≥2 ASCVD events [9]. Information on this validation procedure is provided in the Supplemental Methods and Supplemental Results.

The analyses below were conducted with the imputed NHANES data. We estimated the number and percentage of US adults with a history of ASCVD. Among those with ASCVD, we calculated the percentage with very-high ASCVD risk, LDL-C level ≥ 70 mg/dL, and very-high ASCVD risk with LDL-C ≥ 70 mg/dL. We estimated the percentage of US adults recommended for add-on lipid-lowering therapy among those with a history of ASCVD, very-high ASCVD risk, and LDL-C ≥ 70 mg/dL, separately. We estimated the percentage of US adults receiving add-on lipid-lowering therapy among those with a history of ASCVD, very-high ASCVD risk, and those recommended for add-on lipid-lowering therapy. We repeated these analyses for those taking and not taking statins.

We estimated summary statistics for demographics, health-related characteristics, mean LDL-C, and the distribution of LDL-C levels (<70, 70 to <100, 100 to <130, and ≥130 mg/dL) for all US adults with ASCVD and for those without and with very-high ASCVD risk, separately. Among US adults with very-high ASCVD risk, summary statistics for demographics, health-related characteristics, mean LDL-C, and the distribution of LDL-C levels were estimated for those not recommended and recommended for add-on lipid-lowering therapy, separately. The above analyses were repeated, separately, for adults with a history of CHD, MI, and stroke.

We used Poisson regression models with robust variance estimators to estimate the prevalence ratios for factors associated with (1) LDL-C level ≥ 70 versus <70 mg/dL and (2) being recommended versus not recommended for add-on lipid-lowering therapy. Factors investigated were age, sex, race/ethnicity, current smoking, diabetes, hypertension, CKD, history of heart failure, and statin use. Two levels of adjustment were conducted. The first level of adjustment included age, sex, race/ethnicity and each other variable, one at a time. The second level of adjustment included all variables simultaneously.

In a sensitivity analysis, we estimated the number and percentage of US adults recommended and receiving add-on lipid-lowering therapy without imputing having ≥2 ASCVD events in the NHANES data. For this analysis, very-high ASCVD risk was defined either by having a history of CHD and stroke or by having a history of CHD or stroke and at least two high-risk conditions. We used NHANES sampling weights in all calculations to obtain estimates for the non-institutionalized US adult population, and the survey commands in SAS V14 (SAS Institute, Cary, NC) were used to account for NHANES' multi-stage approach in identifying study participants. The institutional review board at the University of Alabama at Birmingham approved the current study. Informed consent was provided by NHANES and signed by participants in the survey [10].

## 3 Results

### 3.1 US adults with ASCVD recommended for and taking add-on lipid-lowering therapy

**Fig. 1:**
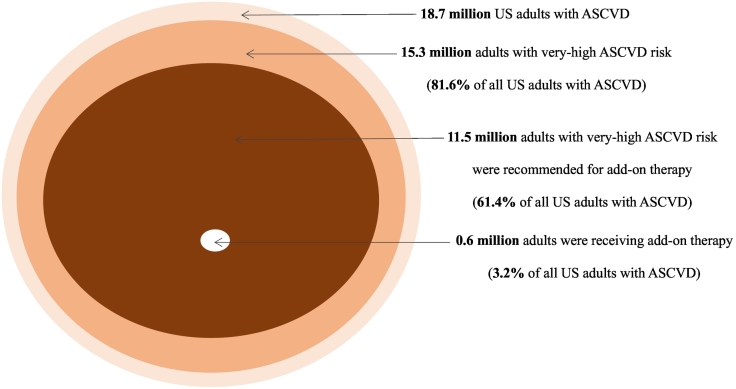
Estimated number and percentage of US adults with ASCVD recommended for and taking add-on lipid-lowering therapy according to the 2018 AHA/ACC multi-society cholesterol guideline Abbreviations: AHA/ACC - American Heart Association/American College of Cardiology, ASCVD - atherosclerotic cardiovascular disease.

An estimated 18.7 million (95 % CI, 16.0–21.4 million) US adults had a history of ASCVD in 2013–2020 (Table 1 and Fig. 1). Among US adults with ASCVD, an estimated 81.6 % (95 % CI, 76.7 %–86.4 %) had very-high ASCVD risk and 60.1 % (95 % CI, 54.5 %–65.7 %) had very-high ASCVD risk and LDL-C ≥ 70 mg/dL. Of US adults with ASCVD, an estimated 61.4 % (95 % CI, 55.8 %–66.9 %) were recommended for add-on lipid-lowering therapy [11.5 million (95 % CI, 9.6–13.3 million)]. Overall, an estimated 3.2 % (95 % CI, 1.2 %–5.3 %) of US adults with ASCVD were taking ezetimibe or a PCSK9 inhibitor, which represented 5.2 % (95 % CI, 1.9 %–8.6 %) of those recommended add-on therapy. The estimated number and percentage recommended and receiving add-on lipid-lowering therapy are presented for US adults with CHD, MI, and stroke, separately, in Supplemental Table 2 and among those taking and not taking a statin, separately, in Supplemental Table 3.

**Table 1:** Estimated percentage of US adults ≥20 years with ASCVD being recommended for add-on lipid-lowering therapy by the 2018 AHA/ACC cholesterol guideline and percentage of US adults receiving add-on lipid-lowering therapy among those recommended.

History of ASCVD [N = 18.7 million (95 % CI, 16.0–21.4 million)]	
Percentage with very-high ASCVD risk	81.6 % (95 % CI, 76.7 % - 86.4 %)^a^
Percentage with LDL cholesterol ≥70 mg/dL	74.9 % (95 % CI, 70.4 % - 79.0 %)^b^
Percentage with LDL cholesterol ≥70 mg/dL and very-high ASCVD risk	60.1 % (95 % CI, 54.5 % - 65.7 %)^c^


Recommended add-on lipid-lowering therapy^d^ [N = 11.5 million (95 % CI, 9.6–13.3 million)]	
Percentage among those with ASCVD	61.4 % (95 % CI, 55.8 % - 66.9 %)
Percentage among those with very-high ASCVD risk	75.3 % (95 % CI, 71.0 % - 79.5 %)
Percentage among those with LDL cholesterol ≥70 mg/dL	80.3 % (95 % CI, 73.2 % - 87.4 %)


Receiving add-on lipid-lowering therapy [N = 0.6 million (95 % CI, 0.2–1.0 million)]	
Percentage among those with ASCVD	3.2 % (95 % CI, 1.2 % - 5.3 %)
Percentage among those with very-high ASCVD risk	3.9 % (95 % CI, 1.5 % - 6.4 %)
Percentage among those recommended add-on lipid-lowering therapy	5.2 % (95 % CI, 1.9 % - 8.6 %)

Abbreviations: AHA/ACC - American Heart Association/American College of Cardiology, ASCVD - atherosclerotic cardiovascular disease,

CI - Confidence Interval, LDL - low-density lipoprotein, PCSK9 - proprotein convertase subtilisin/kexin type-9.

a There were 15.3 million (95 % CI, 13.1–17.5 million) US adults with a history of ASCVD and very-high ASCVD risk.

b There were 14.0 million (95 % CI, 11.8–16.3 million) US adults with a history of ASCVD and LDL cholesterol ≥70 mg/dL.

c There were 11.3 million (95 % CI, 9.5–13.0 million) US adults with a history of ASCVD, LDL cholesterol ≥70 mg/dL and very-high ASCVD risk.

d Recommended add-on lipid-lowering therapy includes those with very-high ASCVD risk and (1) LDL cholesterol ≥70 mg/dL or (2) taking ezetimibe or a PCSK9 inhibitor.

### 3.2 Characteristics of US adults with and without very-high ASCVD risk

The mean age of all US adults with ASCVD was 65.3 years. Overall, 55.5 % were men, and 78.5 %, 12.6 %, and 8.9 % self-reported being of non-Hispanic White, non-Hispanic Black, and Hispanic race/ethnicity, respectively (Table 2). The mean age of US adults without very-high ASCVD risk was 56.8 years compared to 67.2 years for adults with very-high ASCVD risk. Among adults without very-high ASCVD risk, 46.6 % were men, and 75.3 %, 10.7 %, and 14.0 % self-reported being non-Hispanic White, non-Hispanic Black, and Hispanic, respectively. Among US adults with very-high ASCVD risk, 57.5 % were men, and 79.3 %, 13.0 %, and 7.7 % self-reported being non-Hispanic White, non-Hispanic Black, and Hispanic, respectively. Overall, 47.8 % of all adults with ASCVD, 55.2 % and 46.4 % of those without and with very-high ASCVD risk were taking atorvastatin or rosuvastatin, respectively. The characteristics of adults with a history of CHD, MI, and stroke with and without very-high ASCVD risk are presented in Supplemental Tables 4, 5 and 6, respectively.

**Table 2:** Characteristics of US adults ≥20 years with a history of ASCVD with and without very-high ASCVD risk.

Characteristics	Overall (N = 18.7 million)	Without very-high ASCVD risk (N = 3.4 million)	Very-high ASCVD risk		
			Overall (n = 15.3 million)	Not recommended add-on lipid-lowering therapy (n = 3.8 million)	Recommended add-on lipid-lowering therapy (n = 11.5 million)
Age, years, mean (95 % confidence interval)	65.3 (64.2, 66.4)	56.8 (54.1, 59.6)	67.2 (65.8, 68.5)	70.5 (68.6, 72.4)	66.1 (64.7, 67.5)
Age, years, N (%)					
20–54	2.9 (15.7)	1.2 (34.4)	1.8 (11.5)	0.1 (3.7)^a^	1.6 (14.0)
55–64	4.9 (26.2)	1.3 (38.1)	3.6 (23.5)	0.6 (15.3)	3.0 (26.2)
65–74	6.3 (33.4)	0.7 (21.5)	5.5 (36.1)	1.7 (45.8)	3.8 (32.9)
≥75	4.6 (24.7)	0.2 (6.0)^a^	4.4 (28.9)	1.3 (35.1)	3.1 (26.9)
Male, N (%)	10.4 (55.5)	1.6 (46.6)^a^	8.8 (57.5)	2.3 (60.7)	6.5 (56.5)
Race/ethnicity, N (%)					
Non-Hispanic White	13.4 (78.5)	2.5 (75.3)	10.9 (79.3)	2.7 (81.3)	8.2 (78.6)
Non-Hispanic Black	2.1 (12.6)	0.4 (10.7)	1.8 (13.0)	0.4 (11.5)	1.4 (13.5)
Hispanic	1.5 (8.9)	0.5 (14.0)	1.1 (7.7)	0.2 (7.2)	0.8 (7.9)
Smoking, N (%)	3.8 (20.4)	0.3 (9.4)^a^	3.5 (22.9)	0.6 (15.0)	2.9 (25.5)
Diabetes, N (%)	6.9 (37.0)	0.2 (4.4)^a^	6.8 (44.4)	1.8 (47.3)	5.0 (43.4)
Hypertension, N (%)	14.6 (79.1)	1.5 (44.1)	13.1 (86.9)	3.4 (89.1)	9.8 (86.2)
CKD, N (%)	7.0 (37.4)	<0.1 (1.0)	7.0 (45.6)	1.8 (48.9)	5.1 (44.5)
Heart failure, N (%)	3.8 (20.6)	<0.1 (0.8)	3.8 (25.2)	0.9 (23.8)	2.9 (25.6)
Reason for very-high ASCVD risk, N (%)					
Not very-high ASCVD risk	3.45 (18.4)	3.5 (100.0)^a^	0 (0.0)	0 (0.0)	0 (0.0)
One major ASCVD event and multiple risk factors	11.5 (61.3)	0 (0.0)	11.5 (75.2)	2.8 (73.1)	8.7 (75.9)
Two or more major ASCVD events	3.8 (20.2)	0 (0.0)	3.8 (24.8)	1.0 (26.9)	2.8 (24.1)
Statin use, N (%)					
No statins	4.4 (25.6)	1.0 (35.1)	3.4 (23.7)	0.2 (5.4)	3.2 (30.1)
Atorvastatin or Rosuvastatin	8.2 (47.8)	1.5 (55.2)	6.7 (46.4)	2.6 (71.2)	4.0 (37.7)
Other statins	4.6 (26.6)	0.3 (9.7)^a^	4.3 (29.9)	0.9 (23.5)	3.4 (32.2)
Ezetimibe or a PCSK9 inhibitor, N (%)	0.9 (5.0)	0.3 (9.6)^a^	0.6 (3.9)	0 (0.0)	0.6 (5.2)

Abbreviations: ASCVD - atherosclerotic cardiovascular disease, CDC - Centers for Disease Control and Prevention, CKD - chronic kidney disease,

PCSK9 - proprotein convertase subtilisin/kexin type-9.

Numbers in table are mean (95 % confidence interval) for age and number of US adults in million (percentage) for all other variables.

a Estimate does not meet the NHANES recommended threshold for being stable and should be interpreted with caution.

The mean LDL-C was 97.0, 105.5, and 95.1 mg/dL for all US adults with ASCVD and those without and with very-high ASCVD risk, respectively (Table 3). Among adults with very-high ASCVD risk, the mean LDL-C was 54.8 and 108.3 mg/dL for those not recommended and recommended for add-on lipid-lowering therapy, respectively. Among all US adults with ASCVD, 25.1 %, 34.6 %, 23.0 % and 17.4 % had LDL-C of <70, 70 to <100, 100 to <130 and ≥130 mg/dL, respectively. Among adults with very-high ASCVD risk who were recommended for add-on lipid-lowering therapy, 47.2 %, 30.1 % and 20.6 % had LDL-C of 70 to <100, 100 to <130, and ≥130 mg/dL, respectively. The mean and distribution of LDL-C levels for adults with a history of CHD, MI, and stroke with and without very-high ASCVD risk are presented in Supplemental Table 7.

**Table 3:** Estimated mean and distribution of LDL cholesterol in US adults ≥20 years with a history of ASCVD with and without very-high ASCVD risk.

Characteristics	Overall (N = 18.7 million)	Without very-high ASCVD risk (N = 3.4 million)	Very-high ASCVD risk		
			Overall (n = 15.3 million)	Not recommended add-on lipid-lowering therapy (n = 3.8 million)	Recommended add-on lipid-lowering therapy (n = 11.5 million)
LDL cholesterol, mg/dL, mean (95 % CI)	97.0 (93.4, 100.5)	105.5 (97.0, 114.0)	95.1 (91.0, 99.1)	54.8 (51.5, 58.1)	108.3 (104.4, 112.2)
LDL cholesterol, mg/dL, N (%)					
<70	4.7 (25.1)	0.7 (19.8)^a^	4.0 (26.3)	3.8 (100.0)	0.2 (2.1)
70 - <100	6.5 (34.6)	1.1 (30.4)	5.4 (35.5)	0 (0.0)	5.4 (47.2)
100 - <130	4.3 (23.0)	0.8 (24.2)	3.5 (22.7)	0 (0.0)	3.5 (30.1)
≥130	3.3 (17.4)	0.9 (25.6)	2.4 (15.5)	0 (0.0)	2.4 (20.6)

Abbreviations: ASCVD - atherosclerotic cardiovascular disease, CDC - Centers for Disease Control and Prevention, CI - Confidence Interval,

LDL - low-density lipoprotein.

Numbers in the table are mean (95 % confidence interval) or number of US adults in million (percentage).

a Estimate does not meet the NHANES recommended threshold for being stable and should be interpreted with caution.

### 3.3 Factors associated with LDL cholesterol ≥ 70 mg/dL and being recommended for add-on lipid-lowering therapy

An LDL-C ≥ 70 mg/dL was less common among US adults 65–74 years and ≥75 years of age versus 20–54 years of age after adjustment for sex and race/ethnicity (Table 4, left panel). Compared to women, men were less likely to have an LDL-C ≥ 70 mg/dL after adjustment for age and race/ethnicity. An LDL-C ≥ 70 mg/dL was less common among adults taking atorvastatin or rosuvastatin or other statins versus those not taking a statin after adjustment for age, sex, and race/ethnicity. Adults ≥75 years of age versus 20–54 years of age, and adults taking atorvastatin or rosuvastatin or other statins versus not taking a statin were less likely to have an LDL-C ≥ 70 mg/dL, after full multivariable adjustment. After full multivariable adjustment, being recommended versus not recommended for add-on lipid-lowering therapy was more common among current smokers and those with diabetes, hypertension, and CKD, and less common among adults taking atorvastatin or rosuvastatin (Table 4, right panel).

**Table 4:** Factors associated with LDL cholesterol ≥70 mg/dL versus <70 mg/dL (left panel) and recommended versus not recommended for add-on therapy by the 2018 AHA/ACC cholesterol guideline (right panel) in US adults ≥20 years with a history of ASCVD.

Characteristics	LDL-cholesterol ≥70 mg/dL vs <70 mg/dL		Recommended vs not recommended for add-on lipid-lowering therapy	
	Model 1^a^	Model 2^b^	Model 1^a^	Model 2^b^
	Prevalence ratio (95 % CI)	Prevalence ratio (95 % CI)	Prevalence ratio (95 % CI)	Prevalence ratio (95 % CI)
Age, years				
20–54	Reference	Reference	Reference	Reference
55–64	0.92 (0.84–1.02)	1.00 (0.90–1.11)	1.19 (0.97–1.46)	1.16 (0.97–1.38)
65–74	0.81 (0.74–0.90)	0.89 (0.80–1.00)	1.26 (1.06–1.48)	1.18 (1.00–1.39)
≥75	0.78 (0.70–0.87)	0.84 (0.73–0.96)	1.22 (1.02–1.45)	1.16 (0.98–1.38)
Male	0.91 (0.85–0.98)	0.95 (0.88–1.03)	0.95 (0.87–1.04)	0.98 (0.91–1.06)
Race/ethnicity				
White	Reference	Reference	Reference	Reference
Black	0.98 (0.88–1.08)	0.92 (0.82–1.03)	1.02 (0.91–1.15)	0.90 (0.79–1.02)
Hispanic	1.00 (0.91–1.10)	0.95 (0.85–1.06)	0.93 (0.79–1.09)	0.86 (0.73–1.00)
Other	0.95 (0.82–1.10)	0.99 (0.85–1.14)	0.93 (0.78–1.12)	0.93 (0.79–1.10)
Current smoking	1.06 (0.99–1.14)	1.07 (0.98–1.16)	1.33 (1.21–1.47)	1.30 (1.18–1.43)
Diabetes	0.96 (0.88–1.05)	0.99 (0.91–1.09)	1.24 (1.10–1.38)	1.20 (1.07–1.35)
Hypertension	1.02 (0.92–1.12)	1.07 (0.95–1.19)	1.69 (1.38–2.07)	1.50 (1.25–1.80)
CKD	0.99 (0.91–1.07)	1.02 (0.93–1.12)	1.26 (1.14–1.40)	1.16 (1.05–1.29)
Heart failure	0.94 (0.84–1.05)	0.97 (0.87–1.08)	1.16 (1.04–1.31)	1.08 (0.97–1.20)
Statin use				
No statins	Reference	Reference	Reference	Reference
Atorvastatin or Rosuvastatin	0.70 (0.65–0.76)	0.71 (0.65–0.77)	0.72 (0.63–0.82)	0.71 (0.63–0.79)
Other statins	0.85 (0.77–0.95)	0.85 (0.76–0.95)	0.92 (0.79–1.06)	0.88 (0.76–1.01)

Abbreviations: AHA/ACC - American Heart Association/American College of Cardiology, ASCVD - atherosclerotic cardiovascular disease, CI - confidence interval,

CKD - chronic kidney disease, LDL - low-density lipoprotein.

a Includes age, sex, and race/ethnicity and each other factor one at a time.

b Includes all variables simultaneously.

### 3.4 Sensitivity analysis without imputation

In the analysis defining very-high risk as having a CHD and stroke event or a CHD or stroke event with multiple high-risk conditions, an estimated 80.7 % (95 % CI, 75.3 %–85.4 %), 74.9 % (95 % CI, 70.4 %–79.0 %) and 59.6 % (95 % CI, 53.6 %–65.3 %) had very-high ASCVD risk, LDL-C ≥ 70 mg/dL, and very-high ASCVD risk and LDL-C ≥ 70 mg/dL, respectively (Supplemental Table 8). Overall, an estimated 60.8 % (95 % CI, 54.9 %–66.4 %) of US adults with ASCVD were recommended for add-on lipid-lowering therapy, [11.4 million (95 % CI, 9.6–13.2 million)]. Of all US adults with ASCVD, 3.1 % (95 % CI, 1.5 %–5.8 %) were taking ezetimibe or a PCSK9 inhibitor, which represented 5.1 % (95 % CI, 2.4 % - 9.5 %) of those recommended add-on lipid-lowering therapy. Between 62 % and 67 % of US adults with a history of CHD, MI and stroke were estimated to be recommended add-on lipid-lowering therapy and between 2.9 % and 4.0 % of these populations were taking ezetimibe or a PCSK9 inhibitor.

## 4 Discussion

In the current study, we estimated that 18.7 million US adults ≥20 years of age had a history of ASCVD in 2013 to March 2020. A majority of US adults with ASCVD met the 2018 AHA/ACC cholesterol guideline definition of very-high ASCVD risk, with 61.4 % of all US adults with ASCVD being recommended for add-on lipid-lowering therapy by the guideline. However, only 5.2 % of US adults with ASCVD and recommended for add-on lipid-lowering therapy were taking ezetimibe or a PCSK9 inhibitor, and the percentage of US adults taking ezetimibe or a PCSK9 inhibitor was low in all subgroups investigated including those taking and not taking a statin. Also, US adults with high-risk conditions, including current smokers and those with diabetes, hypertension, and CKD, were more likely to be recommended for add-on lipid-lowering therapy, while those taking atorvastatin or rosuvastatin were less likely to be recommended for add-on lipid-lowering therapy by the 2018 AHA/ACC cholesterol guideline.

In the current study of NHANES 2013-March 2020 data, over 80 % of US adults with ASCVD met the 2018 AHA/ACC cholesterol guideline definition of very-high ASCVD risk. This is substantially higher than a previous study of patients with commercial or government health insurance that found 55 % of patients with ASCVD met the guideline definition of very-high ASCVD risk [2]. The difference in the percentage of people with ASCVD meeting the 2018 AHA/ACC cholesterol guideline of very-high risk may be related to the study populations and approaches used for data collection. The current analysis was weighted to provide US nationally representative estimates, while the prior study included individuals with commercial health insurance or government-sponsored supplemental insurance through the Medicare program. As a result, the prior study had a higher percentage of people <65 years of age and relied on administrative claims that may have under-estimated the prevalence of several high-risk conditions, including smoking and CKD [11], [12]. Additionally, the prior study included people with unstable angina and coronary revascularization. Therefore, there could be a substantial number of individuals in the prior study without a history of major ASCVD events and hence, do not meet the 2018 AHA/ACC cholesterol guideline definition of very-high ASCVD risk. Overall, these data suggest a high percentage of people with ASCVD met the 2018 AHA/ACC cholesterol guideline definition of very-high risk in 2013 – March 2020.

### 4.1 Clinical implications

The 2018 AHA/ACC cholesterol guideline recommends add-on lipid-lowering therapy with ezetimibe and/or a PCSK9 inhibitor for adults with very-high ASCVD risk and LDL-C level ≥ 70 mg/dL while taking a maximally tolerated statin dosage [1]. A prior study reported that patients with ASCVD who met the 2018 AHA/ACC cholesterol guideline definition of very-high ASCVD risk had 3 times higher risk for recurrent ASCVD events compared to their counterparts with ASCVD who did not meet the definition of very-high risk [2]. This association was present among all patients and among those taking a high-intensity statin with LDL-C ≥ 70 mg/dL [2]. In the current study, a majority of US adults who met the 2018 AHA/ACC cholesterol guideline definition of very-high ASCVD risk had LDL-C ≥ 70 mg/dL. Given the high prevalence of the very-high ASCVD risk phenotype with LDL-C ≥ 70 mg/dL and the associated increased risk for recurrent events, this population is likely to account for the vast majority of future recurrent ASCVD events [13].

While a majority of US adults with ASCVD were recommended for add-on lipid-lowering therapy, a very small percentage were taking ezetimibe or PCSK9 inhibitor. Prior studies of US adults have reported a low percentage of adults with very high-risk ASCVD to be taking ezetimibe or PCSK9 inhibitor [3], [4], [14]. However, these prior studies were conducted in clinic-based populations and/or among adults with health insurance. The current study extends these prior findings to the general population of US adults with ASCVD and documents a substantial unmet lipid-lowering treatment need.

Randomized controlled trials conducted among adults with ASCVD have shown lipid-lowering medications including statins, ezetimibe, and PCSK9 inhibitors to prevent recurrent ASCVD events [15], [16], [17], [18], [19], [20], [21]. The relative reduction in ASCVD risk with lipid-lowering medications is proportional to the degree to which LDL-C is lowered [22], [23], [24]. Despite these well-established benefits, the use of these medications was low among adults with ASCVD and very-high risk for recurrent events. Results of the current study emphasize the need to identify the reasons for the low use of statins, ezetimibe, and PCSK9 inhibitors among US adults with ASCVD and very-high risk for recurrent events. Many patients report muscle-related side effects as a reason for discontinuing statin therapy [25]. However, most patients who experience statin-related side effects can stay on treatment after re-initiation [26]. Patients and providers have reported the drug approval process and costs as barriers to the initiation and persistence of PCSK9 inhibitors [27], [28], [29]. However, there was a price reduction in PCSK9 inhibitors in 2018. Moreover, under a Markov model, PCSK9 inhibitors are considered to be cost-effective [30]. It is unclear if this information has enhanced uptake of guideline-recommended add-on lipid-lowering therapy. Given the low proportion of US adults recommended for add-on lipid-lowering therapy by the 2018 AHA/ACC cholesterol guideline who are taking ezetimibe or a PCSK9 inhibitor in 2013-March 2020, interventions are likely needed to increase guideline-recommended add-on lipid-lowering therapy among adults with very-high risk for recurrent ASCVD events.

### 4.2 Strengths and limitations

The analysis of NHANES is a major strength of the current study. NHANES includes a stratified population-based sample that can be weighted to produce nationally representative estimates for the non-institutionalized US population. NHANES is carried out by trained study staff and uses rigorous protocols to ensure valid data collection that includes fasting LDL-C and a medication inventory. Despite these strengths, there are potential and known limitations associated with the current study. Due to a modest sample size of NHANES participants with ASCVD, estimates in some sub-groups could be unstable [31]. NHANES did not collect information on having a history of ≥2 ASCVD events. Therefore, we imputed this information using claims data. However, we showed this approach to be valid in an external dataset. Data were only available through March 2020, and it is unclear whether the use of ezetimibe and PCSK9 inhibitors have increased after this period. We were unable to estimate multiple occasions of persistently elevated LDL-C as specified by the 2018 AHA/ACC cholesterol guideline because NHANES collected data at a single time point [6]. Additionally, data were not available on which NHANES participants had experienced statin-associated muscle symptoms or were taking a maximally tolerated statin intensity. However, 70 % of those recommended for add-on lipid-lowering therapy were taking statins and had LDL-C ≥ 70 mg/dL, of which 38 % were taking atorvastatin or rosuvastatin, which are commonly used at high-intensity dosages [32].

## 5 Conclusion

In conclusion, we estimated that a majority of US adults with ASCVD met the 2018 AHA/ACC cholesterol guideline definition of very high risk for recurrent ASCVD events. Additionally, a majority of these individuals were estimated to have LDL-C ≥ 70 mg/dL and were recommended for add-on therapy with ezetimibe or a PCSK9 inhibitor by the 2018 AHA/ACC cholesterol guideline. However, a very low percentage of those recommended for add-on lipid-lowering therapy were taking these medications. The current study highlights a substantial unmet treatment need with add-on lipid-lowering therapy to prevent recurrent ASCVD events.

## CRediT authorship contribution statement

Chibuike J. Alanaeme - conceptualization, methodology, supervision, writing - original draft, visualization, project administration.

Vera Bittner - conceptualization, writing - review & editing.

Todd M. Brown - conceptualization, writing - review & editing.

Lisandro D. Colantonio - conceptualization, methodology, writing - review & editing.

Nafeesa Dhalwani - conceptualization, writing - review & editing, resources.

Jenna Jones - conceptualization, writing - review & editing.

Bethany Kalich - conceptualization, writing - review & editing.

Jason Exter - conceptualization, writing - review & editing.

Elizabeth A. Jackson - conceptualization, writing - review & editing.

Emily B. Levitan - conceptualization, writing - review & editing.

Bharat Poudel - conceptualization, supervision, methodology, writing - review & editing.

Zhixin Wang - data curation, formal analysis.

Mark Woodward - conceptualization, methodology, writing - review & editing.

Paul Muntner - conceptualization, methodology, writing - review & editing, project administration.

Robert S. Rosenson - conceptualization, writing - review & editing.

## Funding

This work was supported by research grant 200709824/7300319916 to the University of Alabama at Birmingham from Amgen Inc. The REGARDS (Reasons for Geographic and Racial Differences in Stroke) study is supported by cooperative agreement U01 NS041588 co-funded by the 10.13039/100000065National Institute of Neurological Disorders and Stroke (NINDS) and the 10.13039/100000049National Institute on Aging (NIA), National Institutes of Health, Department of Health and Human Service. The content is solely the responsibility of the authors and does not necessarily represent the official views of the NINDS or the NIA. Representatives of the NINDS were involved in the review of the manuscript but were not directly involved in the collection, management, analysis or interpretation of the data.

## Declaration of competing interest

The authors declare the following financial interests/personal relationships which may be considered as potential competing interests: Chibuike J. Alanaeme, Bharat Poudel, and Zhixin Wang have nothing to disclose.

Bethany Kalich is an employee of Amgen. Jason Exter was an Amgen employee at the time of the study. Nafeesa Dhalwani and Jenna Jones are employees and stockholders of Amgen.

Todd M. Brown, Lisandro D. Colantonio, and Emily B. Levitan receive research support from 10.13039/100002429Amgen. Paul Muntner receives research support from 10.13039/100002429Amgen and is a consultant to Amgen. Mark Woodward is consultant to Amgen and Freeline.

Vera Bittner has the following disclosures - research support from 10.13039/100002429Amgen, Inc. (Amgen); data and safety monitoring board (DSMB) for Verve Therapeutics; consultant to Pfizer, Inc.; current contract between the University of Alabama at Birmingham (UAB) and Esperion and Novartis; past contract between UAB with Astra Zeneca and DalCor.

Elizabeth A. Jackson has the following disclosures - research support from 10.13039/100002429Amgen and the 10.13039/100000002National Institutes of Health; editorial board of the 10.13039/100000968American Heart Association; consultant to the 10.13039/100005485American College of Cardiology and McKesson Corporation; royalties from Wolters Kluwer (UpToDate); expert witness for Deblase Brown Everly, LLC.

Robert S. Rosenson has the following disclosures - research support from 10.13039/100002429Amgen, Arrowhead, Lilly, Novartis and Regeneron; consulting fees from Amgen, Arrowhead, CRISPR Therapeutics, Precision Biosciences, Novartis, Regeneron, Ultragenyx; honoraria for non-promotional speaking from Amgen, Lilly and Regeneron, royalties from Wolters Kluwer (UpToDate) and has stock holdings in MediMergent, LLC.

## Appendix A Supplementary data

Supplementary material Image 1
