# Supplementary material for: Estimated number and percentage of US adults with atherosclerotic cardiovascular disease recommended add-on lipid-lowering therapy by the 2018 AHA/ACC multi-society cholesterol guideline
Source: Am Heart J Plus. 2022 Aug 27;21:100201. doi: 10.1016/j.ahjo.2022.100201 (PMC10168648; doi:10.1016/j.ahjo.2022.100201)
Supplement: Supplementary file 1 — Supplementary material [file mmc1.docx]

ONLINE SUPPLEMENT ONLY

**Full title**: Estimated number and percentage of US adults with atherosclerotic cardiovascular disease recommended add-on lipid-lowering therapy by the 2018 AHA/ACC multi-society cholesterol guideline

**Supplemental Methods**.

Administrative and claims data from a 5% random sample of Medicare beneficiaries and all individuals in the MarketScan database from 2013 to 2019 were used to estimate the number of participants in the US National Health and Nutrition Examination Survey (NHANES) data with a history of atherosclerotic cardiovascular disease (ASCVD) that had experienced ≥ 2 myocardial infractions (MI) or stroke events during 2013 - March 2020. Medicare is a US government-sponsored health insurance program for adults 65 years of age or older, younger adults with end-stage kidney disease, and who are disabled. MarketScan (Truven Health Analytics, IBM Watson Health, Ann Arbor, Michigan) includes person-level information for about 200 million individuals living in the US with private health insurance or Medicare supplemental insurance. Each individual with a record in the MarketScan and Medicare database between 2013 and 2019 was assigned a random date in this period, referred as an individual’s index date, and the inclusion/exclusion criteria described below were evaluated on this date. We identified 326,053 individuals in the MarketScan database and 416,748 beneficiaries from Medicare with a history of ASCVD on their index date (**Supplemental Figure 2**). We further restricted this study population to those living in the US not including US territories and with continuous medical and pharmacy coverage for the 365 days before their index date. As individuals ≥ 65 years of age may be in both the Medicare and MarketScan databases, we restricted the population in MarketScan to those 21-64 years old, whereas we restricted the population in Medicare to those ≥ 66 years old (i.e., ≥ 65 years old 365 days prior to the index date). After pooling the Medicare and MarketScan data, 399,009 individuals with ASCVD were used for multiple imputation. In the pooled Medicare/MarketScan data, we used the 9th and 10th revisions of the international classification of diseases (ICD-9 and ICD-10, respectively) to identify individuals who had experienced two or more MIs or stroke events prior to their index date as well as other comorbidities chosen to align with those in NHANES. A description for variables created in the Medicare/MarketScan data is provided in **Supplemental Table 9**. Medications were identified using national drug codes and generic names. We appended data from individuals in the Medicare/MarketScan dataset to the NHANES database and used the fully conditional specification method in SAS to perform multiple imputation with 50 data sets, estimating having ≥ 2 ASCVD events or not among NHANES participants with ASCVD. Variables used in the imputation model included calendar year, age, sex, diabetes, hypertension, chronic kidney disease, smoking status, heart failure, and statin use.

To validate the approach described above, we appended the Medicare/MarketScan data to baseline data from the Reasons for Geographic And Racial Differences in Stroke (REGARDS) study, a prospective cohort study that collected information on the self-reported number of MIs and strokes that each participant had experienced. From the baseline REGARDS data (N = 30,239), we included 6,260 participants who completed the study interview and examination, had a history of ASCVD and complete information on triglycerides, and total and high-density lipoprotein cholesterol (HDL-C, **Supplemental Figure 3**). We set REGARDS participants’ information on having ≥ 2 ASCVD events to missing and used the pooled Medicare/MarketScan data described above, age, sex, diabetes, hypertension, chronic kidney disease, smoking status, heart failure, and statin use and the fully conditional specification method in SAS to impute 50 data sets with information on having a history of ≥ 2 ASCVD events. The definition of variables used from the REGARDS study is provided in **Supplemental Table 10**. Results from the validation analysis in REGARDS are provided in the Supplemental Results.

**Supplemental Results**

Among Reasons for Geographic And Racial Differences in Stroke (REGARDS) study participants with atherosclerotic cardiovascular disease (ASCVD), 21.9% (95% CI 20.9% - 23.0%) had two or more ASCVD events (**Supplemental Table 11**). When imputed from the Medicare/MarketScan data, we estimated that 19.6% (95% CI 18.4% - 20.8%) had ≥ 2 ASCVD events. The actual and imputed proportion of REGARDS participants with ASCVD with ≥ 2 ASCVD events was 22.5% and 22.1% among those with LDL-C < 70 mg/dL, respectively, 21.9% and 20.5% among those with LDL-C of 70 to < 100 mg/dL, respectively, 22.2% and 18.9% among those with LDL-C of 100 to < 130 mg/dL and 21.2% and 17.7% among those with LDL-C ≥ 130 mg/dL.

| **Supplemental Table 1**  High-risk conditions for ASCVD as defined in the 2018 AHA/ACC cholesterol guideline ^a^ and adapted to the data available in NHANES. | |
| --- | --- |
| High-risk condition | Definition based on data available in NHANES |
| Age ≥ 65 years | Self-report |
| Diabetes | 1. Hemoglobin A1c ≥ 6.5%, or 2. Self-reported diagnosis of diabetes and concurrent use of insulin or oral glucose-lowering medication. |
| Hypertension | 1. Mean systolic blood pressure ≥ 130 mmHg, 2. Mean diastolic blood pressure ≥ 80 mmHg, or 3. Self-reported diagnosis of high blood pressure and concurrent use of antihypertensive medication. |
| Current smoking | Self-report |
| CKD (eGFR 15-59 mL/min/1.73 m^2^) | CKD (estimated glomerular filtration rate 15–59 ml/min/1.73 m^2^) estimated using the non-race specific CKD-EPI equation. |
| History of congestive heart failure | Self-report |
| Persistently elevated LDL-C (LDL-C ≥100 mg/dL [≥2.6 mmol/L]) despite maximally tolerated statin therapy and ezetimibe | A calculated LDL-C ≥ 100 mg/dL using the Sampson equation:  Calculated LDL-C = (total cholesterol/0.948) – (HDL-C/0.971) – ((triglycerides/8.56) + ((triglycerides*(total cholesterol-HDL-C))/2140) – (triglycerides**2)/16100) – 9.44. |
| Heterozygous familial hypercholesterolemia | Not available |
| History of prior coronary artery bypass surgery or percutaneous coronary intervention outside of the major ASCVD event(s) | Not available |
| Abbreviations: AHA/ACC - American Heart Association/American College of Cardiology, ASCVD - atherosclerotic cardiovascular disease,  CKD - Chronic Kidney Disease, HDL-C - high-density lipoprotein cholesterol, LDL-C - low-density lipoprotein cholesterol.  ^a^ Grundy SM, Stone NJ, Bailey AL, Beam C, Birtcher KK, Blumenthal RS, et al. 2018 AHA/ACC/AACVPR/AAPA/ABC/ACPM/ADA/AGS/APhA/ASPC/NLA/PCNA Guideline on the Management of Blood Cholesterol: A Report of the American College of Cardiology/American Heart Association Task Force on Clinical Practice Guidelines. Circulation 2019;139. https://doi.org/10.1161/CIR.0000000000000625. | |

| **Supplemental Table 2**  Estimated percentage of US adults ≥ 20 years with ASCVD being recommended for add-on lipid-lowering therapy by the 2018 AHA/ACC cholesterol guideline and percentage of US adults receiving add-on lipid-lowering therapy among those recommended in those with a history of CHD, MI, or stroke, separately. | | | |
| --- | --- | --- | --- |
|  | History of CHD | History of MI | History of Stroke |
| Overall | 12.8 million  (95% CI, 10.6 - 15.0 million) | 8.4 million  (95% CI, 6.7 - 10.1 million) | 7.8 million  (95% CI, 6.5 - 9.2 million) |
| Percentage with very-high ASCVD risk | 85.1 % (95% CI, 80.8% - 89.3%) ^a^ | 87.4 % (95% CI, 82.2% - 92.6%) ^d^ | 80.4 % (95% CI, 73.5% - 87.4%) ^g^ |
| Percentage with LDL cholesterol ≥ 70 mg/dL | 72.2 % (95% CI, 67.4% - 76.7%) ^b^ | 75.2 % (95% CI, 68.1% - 81.4%) ^e^ | 77.0 % (95% CI, 67.3% - 85.0%) ^h^ |
| Percentage with LDL cholesterol ≥ 70 mg/dL  and very-high ASCVD risk | 60.7 % (95% CI, 54.7% - 66.7%) ^c^ | 65.5 % (95% CI, 58.1% - 72.9%) ^f^ | 60.5 % (95% CI, 52.8% - 68.2%) ^i^ |
| Recommended add-on lipid-lowering therapy ^j^ | 8.0 million  (95% CI, 6.5 - 9.5 million) | 5.7 million  (95% CI, 4.5 - 6.9 million) | 4.9 million  (95% CI, 3.8 - 6.0 million) |
| Percentage among those with CHD (left column),  MI (middle column), or stroke (right column) | 62.3 % (95% CI, 56.2% - 68.5%) | 67.3 % (95% CI, 60.3% - 74.3%) | 62.7 % (95% CI, 55.3% - 70.1%) |
| Percentage among those with very-high ASCVD risk | 73.3 % (95% CI, 68.1% - 78.4%) | 77.1 % (95% CI, 70.7% - 83.4%) | 78.0 % (95% CI, 70.5% - 85.5%) |
| Percentage among those with LDL cholesterol ≥ 70 mg/dL | 84.1 % (95% CI, 78.5% - 89.7%) | 87.1 % (95% CI, 81.5% - 92.7%) | 78.6 % (95% CI, 68.6% - 88.6%) |
| Receiving add-on lipid-lowering therapy | 0.5 million  (95% CI, 0.1 - 1.0 million) | 0.3 million  (95% CI, 0.0 - 0.6 million) | 0.2 million  (95% CI, 0.0 - 0.5 million) |
| Percentage among those with CHD (left column),  MI (middle column), or stroke (right column) | 4.2% (95% CI, 1.2% - 7.2%) ^k^ | 3.4% (95% CI, 0.0% - 7.2%) ^k^ | 2.9% (95% CI, 0.6% - 8.4%) ^k^ |
| Percentage among those with very-high ASCVD risk | 4.9% (95% CI, 1.5% - 8.4%) ^k^ | 3.9% (95% CI, 0.0% - 8.2%) ^k^ | 3.6% (95% CI, 0.0% - 7.7%) ^k^ |
| Percentage among those recommended for add-on  lipid-lowering therapy | 6.7% (95% CI, 2.0% - 11.4%) ^k^ | 5.1% (95% CI, 0.0% - 10.6%) ^k^ | 4.7% (95% CI, 0.0% - 9.8%) ^k^ |
| Abbreviations: AHA/ACC - American Heart Association/American College of Cardiology, ASCVD - atherosclerotic cardiovascular disease,  CDC - Centers for Disease Control and Prevention, CHD - coronary heart disease, CI - Confidence Interval, LDL - low-density lipoprotein, MI - myocardial infraction.  ^a^ There were 10.9 million (95% CI, 9.1 - 12.7 million) US adults with a history of CHD and very-high ASCVD risk  ^b^ There were 9.3 million (95% CI, 7.7 - 10.8 million) US adults with a history of CHD and LDL cholesterol ≥ 70 mg/dL  ^c^ There were 7.9 million (95% CI, 6.4 - 9.1 million) US adults with a history of CHD, at very-high ASCVD risk and LDL cholesterol ≥ 70 mg/dL  ^d^ There were 7.4 million (95% CI, 5.9 - 8.8 million) US adults with a history of myocardial infarction and very-high ASCVD risk.  ^e^ There were 6.3 million (95% CI, 5.0 - 7.6 million) US adults with a history of myocardial infarction and LDL cholesterol ≥ 70 mg/dL  ^f^ There were 5.5 million (95% CI, 4.4 - 6.6 million) US adults with a history of myocardial infarction, at very-high ASCVD risk and LDL cholesterol ≥ 70 mg/dL  ^g^ There were 6.3 million (95% CI, 5.1 - 7.5 million) US adults with a history of stroke and very-high ASCVD risk.  ^h^ There were 6.0 million (95% CI, 4.7 - 7.4 million) US adults with a history of stroke and LDL cholesterol ≥ 70 mg/dL  ^i^ There were 4.7 million (95% CI, 3.7 - 5.8 million) US adults with a history of stroke, at very-high ASCVD risk and LDL cholesterol ≥ 70 mg/dL  ^j^ Recommended add-on lipid-lowering therapy includes those with very-high ASCVD risk and (1) LDL cholesterol ≥ 70 mg/dL or (2) taking ezetimibe or a proprotein convertase subtilisin/kexin type-9 inhibitor  ^k^ Estimate does not meet the NHANES recommended threshold for being stable and should be interpreted with caution | | | |

| **Supplemental Table 3**  Estimated percentage of US adults ≥ 20 years with ASCVD being recommended for add-on lipid-lowering therapy by the 2018 AHA/ACC cholesterol guideline and percentage of US adults receiving add-on lipid-lowering therapy among those recommended in those taking and not taking statins, separately. | | |
| --- | --- | --- |
|  | Not Taking Statins | Taking Statins |
| History of ASCVD | 4.4 million (95% CI, 3.4 - 5.4 million) | 12.8 million (95% CI, 10.6 - 15.0 million) |
| Percentage with very-high ASCVD risk | 77.7% (95% CI, 65.3% - 90.1%) | 85.8% (95% CI, 81.1% - 90.6%) |
| Percentage with LDL cholesterol ≥ 70 mg/dL | 93.5% (95% CI, 89.5% - 96.3%) | 66.0% (95% CI, 59.9% - 71.8%) |
| Percentage with LDL cholesterol ≥ 70 mg/dL and very-high ASCVD risk | 72.1% (95% CI, 59.7% - 84.6%) | 56.9% (95% CI, 50.0% - 63.7%) |
| Recommended add-on lipid-lowering therapy ^a^ | 3.2 million (95% CI, 2.6 - 3.8 million) | 7.5 million (95% CI, 5.9 - 9.1 million) |
| Percentage among those with ASCVD | 73.2% (95% CI, 60.8% - 85.6%) | 58.4% (95% CI, 51.8% - 65.0%) |
| Percentage among those with very-high ASCVD risk | 94.2% (95% CI, 91.1% - 97.3%) | 68.0% (95% CI, 62.2% - 73.8%) |
| Percentage among those with LDL cholesterol ≥ 70 mg/dL | 77.2% (95% CI, 63.9% - 90.4%) | 86.2% (95% CI, 78.9% - 93.4%) |
| Receiving add-on lipid-lowering therapy | 0.3 million (95% CI, 0.1 - 0.5 million) | 0.3 million (95% CI, 0.0 - 0.7 million) |
| Percentage among those with ASCVD | 6.1% (95% CI, 1.5% - 10.8%) ^b^ | 2.6% (95% CI, 0.0% - 5.2%) ^b^ |
| Percentage among those with very-high ASCVD risk | 7.9% (95% CI, 2.2% - 13.6%) ^b^ | 3.0% (95% CI, 0.0% - 6.0%) ^b^ |
| Percentage among those recommended for add-on lipid-lowering therapy | 8.4% (95% CI, 2.3% - 14.5%) ^b^ | 4.4% (95% CI, 0.0% - 8.9%) ^b^ |
| Abbreviations: AHA/ACC - American Heart Association/American College of Cardiology, ASCVD - atherosclerotic cardiovascular disease, CI - confidence interval,  LDL - low-density lipoprotein, CDC - Centers for Disease Control and Prevention.  ^a^ Recommended add-on lipid-lowering therapy includes those with very-high ASCVD risk and (1) LDL cholesterol ≥ 70 mg/dL or (2) taking ezetimibe or a proprotein convertase subtilisin/kexin type-9 inhibitor.  ^b^ Estimate does not meet the NHANES recommended threshold for being stable and should be interpreted with caution. | | |

| **Supplemental Table 4**  Characteristics of US adults ≥ 20 years with a history of CHD with and without very-high ASCVD risk. | | | | | |
| --- | --- | --- | --- | --- | --- |
| Characteristics | Overall  (N = 12.8 million) | Without very-high ASCVD risk  (N = 1.9 million) | Very-high ASCVD risk | | |
|  |  |  | Overall  (n = 10.9 million) | Not recommended add-on lipid-lowering therapy  (n = 2.9 million) | Recommended add-on lipid-lowering therapy  (n = 8.0 million) |
| Age, years, mean (95% confidence interval) | 66.1 (64.9 - 67.3) | 59.3 (56.3 - 62.3) | 67.4 (65.8 - 68.9) | 69.9 (67.7 - 72.2) | 66.4 (64.9 - 68.0) |
| Age, years, n (%) |  |  |  |  |  |
| 18-54 | 1.5 (11.4) | 0.5 (24.4) | 1.0 (9.1) | 0.1 (4.3) ^a^ | 0.9 (10.9) |
| 55-64 | 3.8 (29.7) | 0.9 (45.5) | 2.9 (26.9) | 0.5 (16.8) ^a^ | 2.4 (30.6) |
| 65-74 | 4.3 (33.8) | 0.4 (23.3) | 3.9 (35.6) | 1.4 (47.0) | 2.5 (31.4) |
| ≥ 75 | 3.2 (25.1) | 0.1 (6.8) | 3.1 (28.3) | 0.9 (31.9) | 2.1 (27.0) |
| Male, n (%) | 8.1 (62.9) | 1.2 (61.4) | 6.9 (63.2) | 2.0 (69.2) | 4.9 (61.0) |
| Race/Ethnicity, n (%) |  |  |  |  |  |
| Non-Hispanic White | 9.3 (72.8) | 1.5 (77.1) | 7.9 (72.1) | 2.1 (73.5) | 5.7 (71.6) |
| Non-Hispanic Black | 1.3 (9.9) | 0.1 (5.5) | 1.2 (10.7) | 0.2 (8.2) | 0.9 (11.6) |
| Hispanics | 1.0 (7.8) | 0.2 (12.5) | 0.8 (7.0) | 0.2 (5.9) | 0.6 (7.4) |
| Other Non-Hispanic | 1.2 (9.4) | 0.1 (4.9) ^a^ | 1.1 (10.2) | 0.4 (12.3) ^a^ | 0.8 (9.5) |
| Smoking, n (%) | 2.6 (20.5) | 0.2 (9.5) ^a^ | 2.4 (22.4) | 0.4 (14.4) | 2.0 (25.4) |
| Diabetes, n (%) | 4.9 (38.2) | 0.1 (7.2) ^a^ | 4.8 (43.6) | 1.4 (48.0) | 3.4 (42.0) |
| Hypertension, n (%) | 10.1 (79.7) | 0.7 (37.3) ^a^ | 9.4 (87.2) | 2.6 (89.1) | 6.8 (86.5) |
| CKD, n (%) | 4.9 (38.3) | <0.1 (0.3) ^a^ | 4.9 (45.0) | 1.3 (46.0) | 3.6 (44.6) |
| Heart failure, n (%) | 3.4 (27.3) | <0.1 (1.4) | 3.4 (31.9) | 0.8 (28.1) | 2.6 (33.3) |
| Reason for very-high ASCVD risk, n (%) |  |  |  |  |  |
| Not very-high ASCVD risk | 1.9 (14.9) | 1.9 (100.0) | 0 (0.0) | 0 (0.0) | 0 (0.0) |
| One major ASCVD event and multiple risk factors | 7.6 (59.6) | 0 (0.0) | 7.6 (70.1) | 2.0 (69.5) | 5.6 (70.3) |
| Two or more major ASCVD events | 3.3 (25.5) | 0 (0.0) | 3.3 (29.9) | 0.9 (30.5) | 2.4 (29.7) |
| Statin use, n (%) |  |  |  |  | 2.0 (26.1) |
| No statins | 2.4 (20.4) | 0.4 (23.0) ^a^ | 2.1 (20.0) | 0.1(4.2) | 3.2 (42.6) |
| Atorvastatin or Rosuvastatin | 6.2 (52.4) | 1.0 (62.6) ^a^ | 5.3 (50.9) | 2.1 (72.3) | 2.3 (31.3) |
| Other statins | 3.2 (27.2) | 0.2 (14.4) ^a^ | 3.0 (29.1) | 0.7 (23.5) | 0.5 (6.7) |
| Ezetimibe or a PCSK9 inhibitor, n (%) | 0.9 (6.8) | 0.3 (17.3) ^a^ | 0.5 (4.9) ^a^ | 0 (0.0) | 2.0 (26.1) ^a^ |
| Abbreviations: ASCVD - atherosclerotic cardiovascular disease, CDC - Centers for Disease Control and Prevention, CHD - coronary heart disease,  CKD - chronic kidney disease, PCSK9 - proprotein convertase subtilisin/kexin type-9.  Numbers in table are mean (95% confidence interval) for age and number of US adults in million (percentage) for all other variables.  ^a^ Estimate does not meet the NHANES recommended threshold for being stable and should be interpreted with caution. | | | | | |

| **Supplemental Table 5**  Characteristics of US adults ≥ 20 years with a history of MI with and without very-high ASCVD risk. | | | | | |
| --- | --- | --- | --- | --- | --- |
| Characteristics | Overall  (N = 8.4 million) | Without very-high ASCVD risk  (N = 1.0 million) | Very-high ASCVD risk | | |
|  |  |  | Overall  (n = 7.4 million) | Not recommended add-on lipid-lowering therapy  (n = 1.7 million) | Recommended add-on lipid-lowering therapy  (n = 5.7 million) |
| Age, years, mean (95% confidence interval) | 64.9 (63.4 - 66.5) | 58.2 (53.8 - 62.6) | 65.9 (64.3 - 67.5) | 69.2 (66.2 - 72.2) | 64.9 (63.1 - 66.7) |
| Age, years, n (%) |  |  |  |  |  |
| 18-54 | 1.1 (13.3) | 0.3 (30.7) | 0.8 (10.8) | 0.1 (4.0) ^a^ | 0.7 (12.9) |
| 55-64 | 2.9 (34.8) | 0.5 (44.9) ^a^ | 2.5 (33.3) | 0.4 (23.3) ^a^ | 2.1 (36.3) |
| 65-74 | 2.4 (28.7) | 0.2 (17.6) | 2.2 (30.3) | 0.7 (38.5) | 1.6 (27.9) |
| ≥ 75 | 2.0 (23.2) | 0.1 (6.8) ^a^ | 1.9 (25.6) | 0.8 (34.2) | 1.3 (23.0) |
| Male, n (%) | 5.8 (68.5) | 0.8 (70.8) ^a^ | 5.0 (68.1) | 1.2 (72.3) | 3.8 (66.9) |
| Race/Ethnicity, n (%) |  |  |  |  |  |
| Non-Hispanic White | 5.9 (70.3) | 0.7 (67.1) | 5.2 (70.7) | 1.1 (63.4) | 4.1 (72.9) |
| Non-Hispanic Black | 0.9 (11.0) | 0.1 (7.0) ^a^ | 0.9 (11.6) | 0.2 (12.6) | 0.6 (11.2) |
| Hispanics | 0.7 (8.4) | 0.2 (17.0) | 0.5 (7.2) | 0.1 (8.5) | 0.4 (6.7) |
| Other Non-Hispanic | 0.9 (10.3) | 0.1 (8.9) ^a^ | 0.8 (10.6) | 0.3 (15.4) ^a^ | 0.5 (9.1) ^a^ |
| Smoking, n (%) | 2.1 (24.8) | 0.2 (17.1) | 1.9 (25.9) | 0.3 (17.1) ^a^ | 1.6 (28.5) |
| Diabetes, n (%) | 3.4 (40.3) | 0.1 (11.1) ^a^ | 3.3 (44.5) | 0.8 (47.6) ^a^ | 2.5 (43.6) |
| Hypertension, n (%) | 6.7 (80.8) | 0.3 (29.2) | 6.4 (88.3) | 1.5 (88.5) | 4.9 (88.3) |
| CKD, n (%) | 3.3 (38.8) | <0.1 (0.6) ^a^ | 3.3 (44.3) | 0.9 (53.4) ^a^ | 2.4 (41.6) |
| Heart failure, n (%) | 2.6 (32.1) | <0.1 (1.2) ^a^ | 2.6 (36.7) | 0.6 (37.5) | 2.1 (36.5) |
| Reason for very-high ASCVD risk, n (%) |  |  |  |  |  |
| Not very-high ASCVD risk | 1.1 (12.6) | 1.1 (100.0) | 0 (0.0) | 0 (0.0) | 0 (0.0) |
| One major ASCVD event and multiple risk factors | 5.0 (59.5) | 0 (0.0) | 5.0 (68.1) | 1.1 (64.9) ^a^ | 3.9 (69.1) |
| Two or more major ASCVD events | 2.3 (27.9) | 0 (0.0) | 2.3 (31.9) | 0.6 (35.1) ^a^ | 1.8 (30.9) |
| Statin use, n (%) |  |  |  |  |  |
| No statins | 1.6 (20.5) | 0.1 (12.3) ^a^ | 1.5 (21.5) | 0.1 (5.5) ^a^ | 1.4 (26.5) |
| Atorvastatin or Rosuvastatin | 3.9 (50.6) | 0.6 (70.8) ^a^ | 3.3 (48.1) | 1.1 (68.5) | 2.2 (41.6) |
| Other statins | 2.2 (28.9) | 0.1 (16.9) ^a^ | 2.1 (30.4) | 0.4 (26.0) | 1.7 (31.8) |
| Ezetimibe or a PCSK9 inhibitor, n (%) | 0.3 (3.7) | 0.03 (2.4) ^a^ | 0.3 (3.9) ^a^ | 0 (0.0) | 0.3 (5.1) ^a^ |
| Abbreviations: ASCVD - atherosclerotic cardiovascular disease, CDC - Centers for Disease Control and Prevention, CKD - chronic kidney disease,  MI - myocardial infarction, PCSK9 - proprotein convertase subtilisin/kexin type-9.  Numbers in table are mean (95% confidence interval) for age and number of US adults in million (percentage) for all other variables.  ^a^ Estimate does not meet the NHANES recommended threshold for being stable and should be interpreted with caution. | | | | | |

| **Supplemental Table 6**  Characteristics of US adults ≥ 20 years with a history of stroke with and without very-high ASCVD risk. | | | | | |
| --- | --- | --- | --- | --- | --- |
| Characteristics | Overall  (N = 7.8 million) | Without very-high ASCVD risk  (N = 1.5 million) | Very-high ASCVD risk | | |
|  |  |  | Overall  (n = 6.3 million) | Not recommended add-on lipid-lowering therapy  (n = 1.4 million) | Recommended add-on  lipid-lowering therapy  (n = 4.9 million) |
| Age, years, mean (95% confidence interval) | 64.5 (62.8 - 66.1) | 53.8 (48.3 - 59.3) | 67.1 (65.2 - 69.0) | 72.0 (69.7 - 74.3) | 65.7 (63.6 - 67.8) |
| Age, years, n (%) |  |  |  |  |  |
| 18-54 | 1.6 (20.6) | 0.7 (46.9) | 0.9 (14.2) | 0.1 (3.8) ^a^ | 0.8 (17.2) |
| 55-64 | 1.7 (21.9) | 0.4 (28.8) ^a^ | 1.3 (20.2) | 0.2 (11.8) ^a^ | 1.1 (22.6) |
| 65-74 | 2.4 (30.9) | 0.3 (19.2) ^a^ | 2.1 (33.7) | 0.5 (38.0) ^a^ | 1.6 (32.5) |
| ≥ 75 | 2.1 (26.7) | 0.1 (5.1) ^a^ | 2.0 (31.9) | 0.6 (46.4) ^a^ | 1.4 (27.8) |
| Male, n (%) | 3.3 (42.7) | 0.4 (28.2) ^a^ | 2.9 (46.2) | 0.6 (42.6) | 2.3 (47.2) |
| Race/Ethnicity, n (%) |  |  |  |  |  |
| Non-Hispanic White | 5.4 (68.9) | 1.0 (63.9) ^a^ | 4.4 (70.1) | 0.9 (68.1) | 3.5 (70.6) |
| Non-Hispanic Black | 1.1 (14.4) | 0.2 (15.9) | 0.9 (14.1) | 0.2 (15.0) | 0.7 (13.8) |
| Hispanics | 0.6 (8.0) | 0.2 (14.2) ^a^ | 0.4 (6.5) | 0.1 (7.8) | 0.3 (6.1) |
| Other Non-Hispanic | 0.7 (8.7) | 0.1 (6.0) ^a^ | 0.6 (9.3) | 0.1 (9.1) | 0.5 (9.4) ^a^ |
| Smoking, n (%) | 1.6 (20.1) | 0.1 (9.2) ^a^ | 1.4 (22.8) | 0.2 (12.9) | 1.3 (25.6) |
| Diabetes, n (%) | 2.7 (34.6) | <0.1 (0.8) ^a^ | 2.7 (42.9) | 0.6 (43.0) | 2.1 (42.9) |
| Hypertension, n (%) | 6.2 (80.7) | 0.8 (53.1) ^a^ | 5.4 (87.1) | 1.2 (88.4) | 4.2 (86.7) |
| CKD, n (%) | 3.0 (37.7) | <0.1 (2.0) ^a^ | 2.9 (46.4) | 0.7 (53.8) | 2.2 (44.3) |
| Heart failure, n (%) | 1.2 (15.1) | 0 (0.0) | 1.2 (18.8) | 0.4 (27.8) | 0.8 (16.3) |
| Reason for very-high ASCVD risk, n (%) |  |  |  |  |  |
| Not very-high ASCVD risk | 1.5 (19.6) | 1.5 (100.0) | 0 (0.0) | 0 (0.0) | 0 (0.0) |
| One major ASCVD event and multiple risk factors | 3.8 (48.9) | 0 (0.0) | 3.8 (60.8) | 0.7 (52.8) ^a^ | 3.1 (63.1) |
| Two or more major ASCVD events | 2.5 (31.5) | 0 (0.0) | 2.5 (39.2) | 0.7 (47.2) ^a^ | 1.8 (36.9) |
| Statin use, n (%) |  |  |  |  |  |
| No statins | 2.4 (33.7) | 0.6 (50.0) ^a^ | 1.8 (30.2) | 0.1 (8.8) ^a^ | 1.7 (36.6) |
| Atorvastatin or Rosuvastatin | 2.6 (35.8) | 0.6 (46.2) ^a^ | 2.0 (33.6) | 0.9 (64.2) ^a^ | 1.1 (24.5) |
| Other statins | 2.2 (30.5) | 0.05 (3.8) ^a^ | 2.1 (36.2) | 0.4 (27.0) | 1.8 (38.9) |
| Ezetimibe or a PCSK9 inhibitor, n (%) | 0.2 (2.9) | 0 (0.0) | 0.2 (3.6) ^a^ | 0 (0.0) | 0.2 (4.7) ^a^ |
| Abbreviations: ASCVD - atherosclerotic cardiovascular disease, CDC - Centers for Disease Control and Prevention, CKD - chronic kidney disease,  PCSK9 - proprotein convertase subtilisin/kexin type-9.  Numbers in table are mean (95% confidence interval) for age and number of US adults in million (percentage) for all other variables.  ^a^ Estimate does not meet the NHANES recommended threshold for being stable and should be interpreted with caution. | | | | | |

| **Supplemental Table 7**  Estimated mean and distribution of LDL cholesterol in US adults ≥ 20 years with a history of CHD, MI and stroke, separately, overall and among those with and without very-high ASCVD risk. | | | | | |
| --- | --- | --- | --- | --- | --- |
|  | Overall | Without very-high ASCVD risk | Very-high ASCVD risk | | |
|  |  |  | Overall | Not recommended add-on  lipid-lowering therapy | Recommended add-on  lipid-lowering therapy |
| History of CHD | 12.8 million | 1.9 million | 10.9 million | 2.9 million | 8.0 million |
| LDL cholesterol, mg/dL, mean (95% CI) | 94.8 (91.0 - 98.6) | 104.7 (89.9 - 119.6) | 93.1 (88.3 - 97.9) | 54.5 (50.0 - 58.9) | 107.2 (102.2 - 112.2) |
| LDL cholesterol, mg/dL, n (%) |  |  |  |  |  |
| <70 | 3.564 (27.8) | 0.439 (22.9) | 3.125 (28.6) | 2.918 (100.0) | 0.206 (2.6) ^a^ |
| 70 - <100 | 4.521 (35.3) | 0.477 (24.9) | 4.044 (37.1) | 0 (0.0) | 4.044 (50.6) |
| 100 - <130 | 2.639 (20.6) | 0.474 (24.8) ^a^ | 2.165 (19.8) | 0 (0.0) | 2.165 (27.1) |
| ≥130 | 2.101 (16.4) | 0.524 (27.3) ^a^ | 1.577 (14.5) | 0 (0.0) | 1.577 (19.7) |
| History of MI | 8.4 million | 1.1 million | 7.4 million | 1.7 million | 5.7 million |
| LDL cholesterol, mg/dL, mean (95% CI) | 96.9 (91.1 - 102.7) | 98.4 (90.2 - 106.5) | 96.7 (90.2 - 103.1) | 52.5 (47.5 - 57.4) | 109.8 (103.2 - 116.4) |
| LDL cholesterol, mg/dL, n (%) |  |  |  |  |  |
| <70 | 2.088 (24.8) | 0.245 (23.1) | 1.843 (25.0) | 1.688 (100.0) | 0.155 (2.7) ^a^ |
| 70 - <100 | 2.929 (34.8) | 0.338 (31.8) | 2.591 (35.2) | 0 (0.0) | 2.591 (45.7) |
| 100 - <130 | 2.029 (24.1) | 0.330 (31.0) ^a^ | 1.699 (23.1) | 0 (0.0) | 1.699 (30.0) |
| ≥130 | 1.376 (16.3) | 0.149 (14.1) | 1.227 (16.7) | 0 (0.0) | 1.227 (21.6) |
| History of stroke | 7.8 million | 1.5 million | 6.3 million | 1.4 million | 4.9 million |
| LDL cholesterol, mg/dL, mean (95% CI) | 99.8 (94.8 - 104.8) | 106.4 (97.3 - 115.5) | 98.2 (92.8 - 103.7) | 55.5 (52.5 - 58.4) | 110.3 (104.3 - 116.3) |
| LDL cholesterol, mg/dL, n (%) |  |  |  |  |  |
| <70 | 1.807 (23.0) | 0.244 (15.9) ^a^ | 1.563 (24.8) | 1.391 (100.0) | 0.172 (3.5) ^a^ |
| 70 - <100 | 2.509 (32.0) | 0.573 (37.2) ^a^ | 1.936 (30.7) | 0 (0.0) | 1.936 (39.3) |
| 100 - <130 | 2.052 (26.1) | 0.360 (23.4) ^a^ | 1.692 (26.8) | 0 (0.0) | 1.692 (34.4) |
| ≥130 | 1.481 (18.9) | 0.361 (23.5) ^a^ | 1.121 (17.8) | 0 (0.0) | 1.121 (22.8) |
| Abbreviations: ASCVD - atherosclerotic cardiovascular disease, CDC - Centers for Disease Control and Prevention, CHD - coronary heart disease,  CI - confidence Interval, LDL - low-density lipoprotein, MI - myocardial infarction.  Numbers in the table are mean (95% confidence interval) or number of US adults in million (percentage).  ^a^ Estimate does not meet the NHANES recommended threshold for being stable and should be interpreted with caution. | | | | | |

| **Supplemental Table 8**  Unimputed percentage of US adults ≥ 20 years with ASCVD being recommended for add-on lipid-lowering therapy by the 2018 AHA/ACC cholesterol guideline and percentage of US adults receiving add-on lipid-lowering therapy among those recommended in those with a history of ASCVD, overall, and history of CHD, MI, or stroke, separately. | | | | |
| --- | --- | --- | --- | --- |
|  | History of ASCVD | History of CHD | History of MI | History of Stroke |
| Overall | 18.7 million  (95% CI, 16.0 - 21.4 million) | 12.8 million  (95% CI, 10.6 - 15.0 million) | 8.4 million  (95% CI, 6.7 - 10.1 million) | 7.8 million  (95% CI, 6.5 - 9.2 million) |
| Percentage with very-high ASCVD risk | 80.7% (95% CI, 75.3% - 85.4%) ^a^ | 84.4% (95% CI, 79.6% - 88.4%) ^d^ | 86.8% (95% CI, 80.5% - 91.7%) ^g^ | 79.6% (95% CI, 71.8% - 86.1%) ^j^ |
| Percentage with LDL cholesterol ≥ 70 mg/dL | 74.9% (95% CI, 70.4% - 79.0%) ^b^ | 72.2% (95% CI, 67.4% - 76.7%) ^e^ | 75.2% (95% CI, 68.1% - 81.4%) ^h^ | 77.0% (95% CI, 67.3% - 85.0%) ^k^ |
| Percentage with LDL cholesterol ≥ 70 mg/dL and very-high ASCVD risk | 59.6% (95% CI, 53.6% - 65.3%) ^c^ | 60.3% (95% CI, 53.9% - 66.4%) ^f^ | 65.1% (95% CI, 57.1% - 72.6%) ^i^ | 59.9% (95% CI, 51.8% - 67.6%) ^l^ |
| Recommended add-on lipid-lowering therapy ^m^ | 11.4 million  (95% CI, 9.6 - 13.2 million) | 7.9 million  (95% CI, 6.5 - 9.4 million) | 5.6 million  (95% CI, 4.4 - 6.8 million) | 4.9 million  (95% CI, 3.8 - 5.9 million) |
| Percentage among those with CHD (left column), MI (middle column),  or stroke (right column) | 60.8% (95% CI, 54.9% - 66.4%) | 61.8% (95% CI, 55.3% - 68.0%) | 67.0% (95% CI, 59.3% - 74.0%) | 62.1% (95% CI, 54.3% - 69.5%) |
| Percentage among those with very-high ASCVD risk | 75.3% (95% CI, 70.7% - 79.5%) | 73.2% (95% CI, 67.6% - 78.4%) | 77.1% (95% CI, 69.9% - 83.4%) | 78.0% (95% CI, 69.3% - 85.1%) |
| Percentage among those with LDL cholesterol ≥ 70 mg/dL | 79.5% (95% CI, 71.2% - 86.3%) | 83.5% (95% CI, 77.1% - 88.7%) | 86.6% (95% CI, 79.9% - 91.8%) | 77.8% (95% CI, 65.9% - 87.1%) |
| Receiving add-on lipid-lowering therapy | 0.6 million  (95% CI, 0.2 – 1.0 million) | 0.5 million  (95% CI, 0.1 - 0.9 million) | 0.3 million  (95% CI, 0.0 - 0.6 million) | 0.2 million  (95% CI, 0.0 - 0.5 million) |
| Percentage among those with CHD (left column), MI (middle column),  or stroke (right column) | 3.1% (95% CI, 1.5% - 5.8%) | 4.0% (95% CI, 1.7% - 8.1%) ^n^ | 3.4% (95% CI, 0.7% - 9.7%) ^n^ | 2.9% (95% CI, 0.6% - 8.4%) ^n^ |
| Percentage among those with very-high ASCVD risk | 3.9% (95% CI, 1.8% - 7.2%) ^n^ | 4.8% (95% CI, 2.0% - 9.5%) ^n^ | 3.9% (95% CI, 0.8% - 11.2%) ^n^ | 3.7% (95% CI, 0.7% - 10.6%) ^n^ |
| Percentage among those recommended add-on lipid-lowering therapy | 5.1% (95% CI, 2.4% - 9.5%) ^n^ | 6.6% (95% CI, 2.7% - 12.9%) ^n^ | 5.1% (95% CI, 1.0% - 14.3%) ^n^ | 4.7% (95% CI, 0.9% - 13.3%) ^n^ |
| Abbreviations: AHA/ACC - American Heart Association/American College of Cardiology, ASCVD - atherosclerotic cardiovascular disease, CDC - Centers for Disease Control and Prevention, CHD - coronary heart disease,  CI - Confidence Interval, LDL - low-density lipoprotein, MI - myocardial infraction.  ^a^ There were 15.1 million (95% CI, 12.9 - 17.3 million) US adults with a history of ASCVD and very-high ASCVD risk.  ^b^ There were 14.0 million (95% CI, 11.8 - 16.3 million) US adults with a history of ASCVD and LDL cholesterol ≥ 70 mg/dL  ^c^ There were 11.2 million (95% CI, 9.4 - 12.9 million) US adults with a history of ASCVD, at very-high ASCVD risk and LDL cholesterol ≥ 70 mg/dL  ^d^ There were 10.8 million (95% CI, 9.0 - 12.6 million) US adults with a history of CHD and very-high ASCVD risk.  ^e^ There were 9.3 million (95% CI, 7.7 - 10.8 million) US adults with a history of CHD and LDL cholesterol ≥ 70 mg/dL  ^f^ There were 7.7 million (95% CI, 6.4 - 9.1 million) US adults with a history of CHD, at very-high ASCVD risk and LDL cholesterol ≥ 70 mg/dL  ^g^ There were 7.3 million (95% CI, 5.9 - 8.7 million) US adults with a history of myocardial infarction and very-high ASCVD risk.  ^h^ There were 6.3 million (95% CI, 5.0 - 7.6 million) US adults with a history of myocardial infarction and LDL cholesterol ≥ 70 mg/dL  ^i^ There were 5.5 million (95% CI, 4.4 - 6.6 million) US adults with a history of myocardial infarction, at very-high ASCVD risk and LDL cholesterol ≥ 70 mg/dL  ^j^ There were 6.3 million (95% CI, 5.1 - 7.4 million) US adults with a history of stroke and very-high ASCVD risk.  ^k^ There were 6.0 million (95% CI, 4.7 - 7.4 million) US adults with a history of stroke and LDL cholesterol ≥ 70 mg/dL  ^l^ There were 4.7 million (95% CI, 3.7 - 5.7 million) US adults with a history of stroke, at very-high ASCVD risk and LDL cholesterol ≥ 70 mg/dL  ^m^ Recommended add-on lipid-lowering therapy includes those with very-high ASCVD risk and (1) LDL cholesterol ≥ 70 mg/dL or (2) taking ezetimibe or a proprotein convertase subtilisin/kexin type-9 inhibitor.  ^n^ Estimate does not meet the NHANES recommended threshold for being stable and should be interpreted with caution. | | | | |

| **Supplemental Table 9**  Variables included in the MarketScan/Medicare cohort. | |
| --- | --- |
| Variables | Variable definition |
| Age | Age on index date |
| Sex | Male or female |
| Smoking status | 1. ≥1 hospitalization with a discharge diagnosis code of tobacco use 2. ≥1 physician evaluation and management visit with a diagnosis code of tobacco use 3. ≥1 hospitalization with a discharge diagnosis code or physician evaluation and management visit of tobacco use 4. ≥1 pharmacy claim for nicotine or varenicline |
| Diabetes | Any of the following using all claims prior to the index date through the index date, inclusive:   1. At least 1 inpatient claim with a discharge ICD-9 or ICD-10 diagnosis 2. At least 2 carrier claims, carrier line or outpatient claims with ICD-9 or ICD-10 diagnoses, linked by CLAIM_ID to an ambulatory physician evaluation and management claim, with the 2 claims occurring at least 7 days apart 3. At least 1 pharmacy claim for an oral antidiabetic drug fill or insulin |
| Myocardial infraction (MI) | Episodes of acute MI or Old MI or subsequent episode of care for MI prior to the index date |
| Ischemic stroke | An inpatient claim with a discharge diagnosis code for ischemic stroke in the primary discharge diagnosis position any time prior to index date. To be defined as an ischemic stroke event, the secondary diagnosis codes cannot be after a primary diagnosis code for ischemic stroke. |
| History of atherosclerotic cardiovascular disease (ASCVD) | Defined as having a history of MI or ischemic stroke as defined above |
| Chronic kidney disease (CKD) | Any of the following using all available claims prior to the index date through the index date, inclusive:   1. ≥1 inpatient claim with a discharge diagnosis code of chronic kidney disease in any discharge diagnosis position. 2. ≥1 physician evaluation and management visit with a diagnosis code of chronic kidney disease in any position. 3. For Medicare data, if the flag ESRD_IND in the Master beneficiary summary file is checked then the participant will be categorized as having a history of CKD. |
| Heart failure | Any of the following using all available claims prior to the index date through the index date:   1. ≥ 1 inpatient claim with ICD-9 or 10 diagnoses (any position) 2. ≥ 2 outpatient or carrier claims on separate calendar days with ICD-9 or 10 diagnoses (any position), linked (by CLAIM_ID in Medicare) to an ambulatory physician evaluation and management claim. |
| Hypertension | Any of the following:   1. ≥1 inpatient claim with an ICD-9 or 10 discharge diagnosis code in any discharge diagnosis position. 2. ≥2 physician evaluation and management visits (i.e., outpatient or carrier claim) claims with an ICD-9 or an ICD-10 diagnosis code of in any position at least 30 days apart. 3. Two or more pharmacy fills for an antihypertensive medication |
| Statin use | Defined as 3 categories of statin use as defined below:   1. No statin use will be defined by having no prescription fill for any statin dose and type prior to the index date, inclusive. 2. Use of low/moderate-intensity statin will be defined by ≥1 prescription fills for a low/moderate-intensity statin with no high-intensity statin fills prior to the index date, inclusive. 3. Use of high-intensity statin will be defined by ≥1 prescription fills for any high-intensity statin prior to the index date, inclusive. High-intensity statin includes atorvastatin 40-80 mg, rosuvastatin 20-40 mg and simvastatin 80 mg. |
| Persistently elevated low-density lipoprotein cholesterol (LDL-C) | To be defined as having persistently elevated LDL-C, the most proximal LDL-cholesterol must be ≥ 100 mg/dL despite maximally tolerated statin and ezetimibe. For patients with ≥2 LDL-cholesterol values available, they are required to meet any of the following criteria:   - 2 consecutive LDL-cholesterol ≥ 100 mg/dL, - Had a statin or ezetimibe prescription filled prior to the most proximal LDL-cholesterol value. |
| Very high ASCVD risk | Defined as having a history of two or more major ASCVD events or 1 major ASCVD events plus ≥ 2 high-risk conditions. |
| High-risk conditions | Age ≥65 years  Diabetes (defined above)  Hypertension (defined above)  CKD (estimated glomerular filtration rate 15–59 ml/min/1.73 m^2^)  Current smoking (defined above)  Persistently elevated LDL-C (≥100 mg/dl [≥2.6 mmol/l]) and taking a statin and ezetimibe  History of heart failure (defined above). |
| Abbreviations: ICD-9 - International Classification of Diseases 9th Revision, ICD-10 - International Classification of Diseases 10th Revision. | |

| **Supplemental Table 10**  Variables included in the REGARDS cohort. | |
| --- | --- |
| Age | Self-report |
| Sex | Self-report |
| Smoking Status | Self-report |
| Diabetes | 1. Hemoglobin A1c ≥ 6.5% 2. Self-reported diagnosis of diabetes and concurrent use of insulin or oral 3. glucose-lowering medication. |
| History of CHD | History of heart disease (self-reported myocardial infarction, CABG, bypass, angioplasty, or stenting or evidence of MI via ECG) |
| Stroke | Self-report |
| History of ASCVD | Having a history of CHD, or stroke as defined above. |
| History of 2 or more ASCVD | Defined as having any of the following:   - history of CHD and Stroke - 2 or more MI events - 2 or more stroke events |
| Hypertension | 1. Mean systolic blood pressure ≥ 130 mmHg. 2. Mean diastolic blood pressure ≥ 80 mmHg. 3. Self-reported diagnosis of high blood pressure and concurrent use of antihypertensive medication. |
| CKD | eGFR < 60 mL/min/1.73 m^2^ or albumin-to-creatinine ratio > 30 mg/g |
| Heart failure | Yes/No based on heart failure specific medication use |
| Statin use | Yes/No |
| Persistently elevated LDL-cholesterol | Having calculated LDL-cholesterol ≥ 100 mg/dL using the following Sampson equation:  Calculated LDL cholesterol = (total cholesterol/0.948) – (HDL/0.971) – ((triglycerides/8.56) + ((triglycerides*(total cholesterol-HDL))/2140) – (triglycerides**2)/16100) – 9.44. |
| Very high ASCVD risk | Defined as the following:   1. Having a history of two or more ASCVD events (i.e., a history of CHD and stroke or ≥ 2 MI events or ≥ 2 stroke events, as defined above), Or Having ≥ 2 of the high-risk conditions below. |
| High-risk conditions: | Age ≥65 years  Diabetes  Hypertension  CKD (estimated glomerular filtration rate 15–59 ml/min/1.73 m^2^)  Current smoking  Persistently elevated LDL-C (≥100 mg/dl [≥2.6 mmol/l]) taking a statin  History of heart failure. |
| Abbreviations: ASCVD - atherosclerotic cardiovascular disease, CABG - coronary artery bypass graft, CHD - coronary heart disease, ECG – electrocardiogram,  eGFR - estimated glomerular filtration rate, HDL - high-density lipoprotein, LDL - low-density lipoprotein, MI - myocardial infraction,  REGARDS - Reasons for Geographic And Racial Differences in Stroke, | |

| **Supplemental Table 11**  Actual and imputed prevalence of a history of ≥ 2 ASCVD events in the REGARDS study. | | | | |
| --- | --- | --- | --- | --- |
| Characteristics | REGARDS Participants with ASCVD | REGARDS Participants with ≥2 ASCVD events | Actual REGARDS data  ≥2 ASCVD events | Imputed REGARDS data  ≥2 ASCVD events |
|  | N (%) | N (%) | Prevalence (95% CI) | Prevalence (95% CI) |
| Overall | 6,260 | 1,372 | 21.9 (20.9 - 23.0) | 19.6 (18.4 - 20.8) |
| Age, years |  |  |  |  |
| 45-54 | 366 (5.9) | 75 (5.5) | 20.5 (16.7 - 25.1) | 13.5 (9.8 - 18.5) |
| 55-64 | 1,884 (30.1) | 415 (30.2) | 22.0 (20.2 - 24.0) | 16.4 (14.5 - 18.5) |
| 65-74 | 2,334 (37.3) | 526 (38.3) | 22.5 (20.9 - 24.3) | 20.2 (18.4 - 22.3) |
| ≥ 75 | 1,676 (26.8) | 356 (25.9) | 21.2 (19.4 - 23.3) | 23.5 (20.9 - 26.3) |
| Sex |  |  |  |  |
| Male | 3,654 (58.4) | 841 (61.3) | 23.0 (21.7 - 24.4) | 19.9 (18.4 - 21.6) |
| Female | 2,606 (41.6) | 531 (38.7) | 20.4 (18.9 - 22.0) | 19.0 (17.3 - 21.0) |
| Race/Ethnicity |  |  |  |  |
| White | 3,832 (61.2) | 767 (55.9) | 20.0 (18.8 - 21.3) | 18.3 (16.8 - 19.9) |
| Black | 2,428 (38.8) | 605 (44.1) | 24.9 (23.3 - 26.7) | 21.5 (19.6 - 23.6) |
| Current smoking | 1,019 (16.3) | 278 (20.3) | 27.3 (24.7 - 30.2) | 21 (18.2 - 24.3) |
| Diabetes | 2,025 (32.4) | 536 (39.1) | 26.5 (24.6 - 28.5) | 25.5 (23.3 - 28.0) |
| Hypertension | 4,595 (73.4) | 1,095 (79.8) | 23.8 (22.6 - 25.1) | 21.9 (20.5 - 23.3) |
| CKD | 2,189 (35.0) | 581 (42.3) | 26.5 (24.8 - 28.5) | 25.9 (23.7 - 28.2) |
| Heart failure | 1,480 (23.6) | 457 (33.3) | 30.9 (28.6 - 33.3) | 29 (26.2 - 32.1) |
| Statin Use | 3,429 (54.8) | 829 (60.4) | 24.2 (22.8 - 25.7) | 22 (20.4 - 23.7) |
| LDL-cholesterol, mg/dL |  |  |  |  |
| <70 | 783 (12.5) | 176 (12.8) | 22.5 (19.7 - 25.6) | 21.4 (17.9 - 25.6) |
| 70 - <100 | 2,231 (35.6) | 488 (35.6) | 21.9 (20.2 - 23.7) | 20.5 (18.6 - 22.7) |
| 100 - <130 | 1,888 (30.2) | 420 (30.6) | 22.2 (20.4 - 24.2) | 18.9 (16.9 - 21.2) |
| ≥130 | 1,358 (21.7) | 288 (21.0) | 21.2 (19.1 - 23.5) | 17.7 (15.3 - 20.5) |
| Abbreviations: ASCVD - atherosclerotic cardiovascular disease, CI - confidence interval, CKD - chronic kidney disease, LDL - Low-density Lipoprotein,  REGARDS - Reasons for Geographic And Racial Differences in Stroke. | | | | |

Supplemental Figure 1. Selection of NHANES participants with a history of ASCVD during 2013 – March 2020 for the current analysis

All participants in NHANES 2013-2104, 2015-2016, and 2017- March 2020

N = 35,706

Participants who completed the study interview and examination

N = 33,657

Participants at least 20 years of age

N = 19,606

Participants who attended a morning session

N = 9,518

Participants who fasted 8 to 24 hours before their examination

N = 8,461

Participants who had a history of ASCVD

N = 818

Participants with information on triglycerides, total cholesterol, and HDL-cholesterol

N = 805

Abbreviations: ASCVD - atherosclerotic cardiovascular disease, CKD - chronic kidney disease, HDL - high-density lipoprotein cholesterol,

NHANES - National Health and Nutrition Examination Survey.

Supplemental Figure 2. Flow chart for the selection of Marketscan/Medicare beneficiaries for imputing a history of ≥ 2 ASCVD events.

Marketscan individuals with a history of ASCVD at any time prior to their randomly-selected index date

N = 526,053

5% random sample of Medicare beneficiaries with a history of ASCVD at any time prior to their randomly-selected index date

N = 416,748

Individuals who had continuous fee-for-service inpatient, outpatient and pharmacy coverage and were living in the US (excluding US territories) for 365 days prior to their index date through their index date

N = 291,028

Beneficiaries who had continuous fee-for-service inpatient, outpatient and pharmacy coverage and were living in the US (excluding US territories) for 365 days prior to their index date through their index date

N = 215,839

Individuals from MarketScan who were 21 - 64 years (N = 256,235) on their index date, and beneficiaries from Medicare ≥ 66 years of age on their index date (N = 142,774)

N = 399,009

Imputation was based on calendar period, age, sex, diabetes, hypertension, history of CKD, current smoking, heart failure, and statin use.

Abbreviations: ASCVD - atherosclerotic cardiovascular disease, CKD - chronic kidney disease, NHANES - National Health and Nutrition Examination Survey.

Supplemental Figure 3. Flow chart for the selection of participants from the Reasons for Geographic And Racial Differences in Stroke (REGARDS) study to validate the imputation of a history of ≥ 2 ASCVD events.

All participants in REGARDS data

N = 30,239

Participants who completed the study interview and examination

N = 30,138

Participants with ASCVD

N = 6,624

Participants with information on triglycerides, total cholesterol and HDL cholesterol

N = 6,260

Imputation was based on calendar period, age, sex, diabetes, hypertension, CKD, current smoking, heart failure, and statin use.

Abbreviations: ASCVD - atherosclerotic cardiovascular disease, CKD - Chronic Kidney Disease, HDL - high density lipoprotein,

NHANES - National Health and Nutrition Examination Survey, REGARDS - Reasons for Geographic And Racial Differences in Stroke.
